# Essential oils purified from Schisandrae semen inhibits tumor necrosis factor-α-induced matrix metalloproteinase-9 activation and migration of human aortic smooth muscle cells

**DOI:** 10.1186/s12906-015-0523-9

**Published:** 2015-02-05

**Authors:** Jin-Woo Jeong, Joo Wan Kim, Sae Kwang Ku, Sung Goo Kim, Ki Young Kim, Gi-Young Kim, Hye Jin Hwang, Byung Woo Kim, Hae Young Chung, Cheol Min Kim, Yung Hyun Choi

**Affiliations:** Department of Biochemistry, Dongeui University College of Korean Medicine, Busan, 614-052 Republic of Korea; Research Institute, Bio-Port Korea INC, Marine Bio-industry Development Center, Busan, 619-912 Republic of Korea; Department of Anatomy and Histology, College of Korean Medicine, Daegu Haany University, Gyeongsan, 712-715 Republic of Korea; Laboratory of Immunobiology, Department of Marine Life Sciences, Jeju National University, Jeju, 690-756 Republic of Korea; Anti-Aging Research Center & Blue-Bio Industry RIC, Dongeui University, Busan, 614-714 Republic of Korea; Department of Food and Nutrition, Dongeui University, Busan, 614-714 Republic of Korea; Department of Life Science and Biotechnology, Dongeui University, Busan, 614-714 Republic of Korea; Molecular Inflammation Research Center for Aging Intervention (MRCA), Department of Pharmacy, Pusan National University, Busan, 609-735 Republic of Korea; Department of Biochemistry, Busan National University College of Medicine, Yangsan, 626-870 Republic of Korea

**Keywords:** Schisandrae semen essential oil, HASMCs, Invasion, MMP-9, NF-κB

## Abstract

**Background:**

The migration of vascular smooth muscle cells from the tunica media to the subendothelial region may be a key event in the development of atherosclerosis after arterial injury. In this study, we investigated the potential mechanisms underlying the anti-atherosclerotic effects of Schisandrae Semen essential oil (SSeo) in human aortic smooth muscle cells (HASMCs).

**Methods:**

Metalloproteinase-2/9 (MMP-2/9) activity was evaluated by gelatin zymography and gelatinase activity assay kit. The possible mechanisms underlying SSeo-mediated reduction of by tumor necrosis factor (TNF)-α-induced cell invasion and inhibition of secreted and cytosolic MMP-9 production in HASMCs were investigated.

**Results:**

Our results indicate that SSeo treatment has an inhibitory effect on activation as well as expression of MMP-9 induced by TNF-α in HASMCs in a dose-dependent manner without significant cytotoxicity. SSeo attenuated nuclear translocation of TNF-α-mediated nuclear factor-kappa B (NF-κB) and blocked degradation of the NF-κB inhibitor proteins as well as the production of reactive oxygen species. SSeo also reduced TNF-α-induced production of pro-inflammatory mediators such as nitric oxide and prostaglandin E_2_ and inhibited inducible nitric oxide synthase and cyclooxygenase-2 expression in HASMCs. Furthermore, the Matrigel migration assay showed that SSeo effectively reduced TNF-α-induced HASMC migration compared with that in the control group.

**Conclusions:**

Taken together, these results suggest that SSeo treatment suppresses TNF-α-induced HASMC migration by selectively inhibiting MMP-9 expression, which was associated with suppression of the NF-κB signaling pathway. Taken together, these results suggest that SSeo has putative potential anti-atherosclerotic activity.

## Background

Proliferation and migration of vascular smooth muscle cells (VSMCs) from the tunica media to the subendothelial region play a major role in the development and progression of atherosclerosis, which is a progressive pathological disorder that often leads to cardiovascular and cerebrovascular diseases. During the early stages of atherosclerosis or arterial wall injury, VSMCs migrate to the intimal layer of the arterial wall, causing intimal thickening [1,2]. Accumulating evidence indicates that activation of matrix metalloproteinases (MMPs) may contribute to the pathogenesis of atherosclerosis by facilitating migration of VSMCs through degradation or remodeling of the extracellular matrix (ECM) surrounding cells [3-5]. Among MMPs, gelatinase MMP-9 is particularly critical for the development of arterial lesions *via* its regulation of both VSMC migration and proliferation in the pathogenesis of atherosclerosis [6-8]. The cytokine tumor necrosis factor (TNF)-α secreted by VSMCs accumulates in atherosclerotic lesions and induces marked proliferation and migration of VSMCs [8,9]. The synthesis and secretion of MMP-9, of which basal levels are usually low in VSMCs, but not MMP-2, can be stimulated by a variety of stimuli including growth factors and cytokines such as TNF-α through activation of a transcription factor nuclear factor-kappa B (NF-κB) [10-13]. NF-κB is normally present in the cytosol in an inactive state through interaction with inhibitor of NF-κB (IκB) proteins. The NF-κB dimer dissociates from IκB and translocates to the nucleus following inflammatory or other stimuli that leads to degradation of the IκB protein. In the nucleus, NF-κB binds to promoter regions and induces the expression of a wide variety of genes including various inflammatory factors, adhesion molecules, and MMPs [14,15].

Although the relative contribution of reactive oxygen species (ROS) and inflammatory mediators in the vasculature remains ambiguous, they integrate cellular signaling pathways involved in VSMC proliferation and migration associated with atherosclerosis. Under normal physiological conditions, ROS quenching by antioxidant enzymes is sufficient to maintain the restitution of antioxidant/pro-oxidant equilibrium following an oxidative challenge [16,17]. However, when the production of ROS exceeds endogenous antioxidant capacity, oxidative stress results in abnormal physiological responses, with subsequent severe damage to proteins, lipids, and DNA. In addition, inflammatory factors also play important roles stimulating localized pathological process in atherogenesis [18-20]. Furthermore, oxidative stress can also activate or increase the expression of redox-sensitive genes, including pro-inflammatory factors and MMPs, through activation of the NF-κB signaling pathway [21,22].

Essential oils, which are complex mixtures of volatile compounds produced by aromatic plants, show various pharmacological effects such as antioxidant, antimicrobial and antiseptic effects [23,24]. Plant essential oils exert beneficial effects on various smooth muscle disorders [25-33]. Schisandrae fructus [*Schisandra chinensis* (Turcz.) Baillon] is a medicinal herb widely used to treat various inflammatory and immune diseases, central nervous and cardiovascular disorders, hypertension, and blood sugar and acid–base balance in East Asian countries such as Korea, Taiwan, Japan, China, and Russia [34,35]. According to recent research, essential oil extracted from Schisandrae fructus has been found to have pharmacological activities such as antibacterial and antioxidant activities [36-39]. However, the underlying molecular mechanisms of the potential anti-atherosclerosis effects of Schisandrae semen have not yet been elucidated, particularly with respect to the inhibitory activity of MMPs and migration in VSMCs. Therefore, in the present study, Schisandrae semen essential oil (SSeo) was examined for its potential anti-atherosclerotic effects in human aortic smooth muscle cells (HASMCs). We provide evidence showing that SSeo suppressed TNF-α-induced MMP-9 expression by inhibiting MMP-9 gene transcription. Additionally, suppression of HASMC migration by SSeo appeared to block MMP-9 expression and intracellular ROS accumulation by inhibiting the NF-κB signal pathway.

## Methods

### Preparation of SSeo

Reddish brown clear SSeo was prepared by maceration and hydrodistillation methods as follows. Briefly, dried seeds of *S. chinensis* (Turcz.) Baillon were collected around Mungyeong-city (Gyeongbuk, Republic of Korea) on October 2013 and completely dried at 180°C in a furnace (Daihan Scientific Co., Seoul, Republic of Korea). A voucher specimen (accession number DSSC-1) was deposited at the Medical Research Center for Globalization of Herbal Formulation of Daegu Haany University. The dried seeds were then pulverized and lyophilized in a programmable freeze dryer (Freezone 1; Labconco Co., Kansas City, MO, USA). Lyophilized materials were extracted with 100% ethanol by maceration at room temperature for 24 h, filtered, and then concentrated using a rotary vacuum evaporator (Buchi Rotavapor R-144, BÜCHI Labortechnik, Flawil, Switzerland). Finally, the SSeo (Lot. 2012KuSSeo) was isolated by hydrodistillation using a Clevenger-type apparatus for 3 h according to the method recommended in a previous study [40]. The oil was stored in a refrigerator at 4°C to protect from light and degeneration. The yield of the oil based on the dried weight of the Schisandrae Semen was 0.66%.

### Cell culture

HASMCs originating from normal human tissue were obtained from Bio-Whittaker (Walkersville, MD, USA). They were cultured in SMC growth medium-2 (Gibco-BRL, Grand Island, NY, USA) containing 10% fetal bovine serum (FBS), 2 ng/ml human basic fibroblast growth factor, 0.5 ng/ml human epidermal growth factor, 50 μg/ml gentamicin, 50 μg/ml amphotericin-B, and 5 μg/ml bovine insulin at 37°C, in a humidified atmosphere of 5% CO_2_ and 95% air. All experiments were performed with HASMCs from passages 7–13.

### Cell viability assay

The cytotoxic effect of SSeo and TNF-α on HASMCs was investigated using the 3-(4,5-dimethylthiazol-2-yl)-2,5-diphenyl tetrazolium bromide (MTT) assay. Briefly, the cells were plated at 5 × 10^3^ cells/well in 96-well culture plates and allowed to attach for 24 h. The cells were either treated or not treated with different concentrations of SSeo for 1 h, and then 100 ng/mL TNF-α was added. After a 24 h incubation, MTT solution (0.5 mg/ml, Sigma-Aldrich Chemical Co., St. Louis, MO, USA) was added to each well containing conditioned media and incubated for another 3 h at 37°C. Then, the medium was removed and dimethyl sulfoxide (DMSO) was added to each well. After shaking, the absorbance of the solubilized blue formazan was measured at 540 nm with a microplate reader (Dynatech MR-7000; Dynatech Laboratories, Chantilly, VA, USA) and results were expressed as cell viability relative to the untreated control, which were considered 100% viable.

### Gelatin zymography

The gelatinolytic activities of MMP-2 and MMP-9 in the conditioning culture medium were assayed by electrophoresis on 10% polyacrylamide gels containing 1 mg/mL gelatin at 4°C. After electrophoresis, the gels were washed in 2.5% Triton X-100 for 1 h and incubated at 37°C for 24 h in activation buffer (50 mM Tris–HCl, pH 7.5, 150 mM NaCl, 10 mM CaCl_2_, and 0.02% NaN_3_). After staining with Coomassie Blue R-250 (10% glacial acetic acid, 30% methanol, and 1.5% Coomassie Brilliant Blue; Invitrogen Co., Carlsbad, CA, USA) for 2 h, the gels were destained with a solution of 10% glacial acetic acid and 30% methanol without Coomassie Blue for 1 h. White lysis zones, indicating gelatin degradation, were revealed by staining with Coomassie Brilliant Blue R-250 [41].

### *In vitro* MMP activity assay

MMP activity in the supernatant was also measured using the MMP Gelatinase Activity Assay Kit (Chemicon International Inc., Temecula, CA, USA), according to the manufacturer’s instructions. Briefly, aliquots of culture media were incubated with biotinylated gelatinase substrates provided by the manufacturer to cleave active MMP-2 and MMP-9 in the culture media. The fragments were then added to a biotin-binding 96-well plate and incubated for 30 min at 37°C to allow the biotin-containing fragments to bind to the plate while digestion continued. The digested but unbound fragments were removed by repeated washing, whereas the undigested biotin-labeled gelatinase that bound to the plate was detected by adding a streptavidin–enzyme complex that resulted in a colored product measured at a wavelength of 540 nm with a microplate reader.

### RNA isolation and reverse transcriptase-polymerase chain reaction (RT-PCR)

Total RNA was isolated using TRIzol reagent (Invitrogen Co.) according to the manufacturer’s protocol, and 2 μg of RNA was used for cDNA synthesis using M-MLV reverse transcriptase (Promega, Madison, WI, USA). RT-generated cDNA encoding MMP-2, MMP-9, tissue inhibitors of metalloproteinase (TIMP)-1, TIMP-2, inducible nitric oxide synthase (iNOS) and cyclooxygenase-2 (COX-2) genes was amplified by PCR using specific primers, which were purchased from Bioneer (Seoul, Republic of Korea). The PCR primers were as follows: MMP-9 (5′-CGG AGC ACG GAG ACG GGT AT-3′ and 5′-TGA AGG GGA AGA CGC ACA GC-3′), MMP-2 (5′-CCC CTA TCT ACA CCT ACA CCA AGA AC-3′ and 5′-CCC CTA TCT ACA CCT ACA CCA AGA AC-3′), TIMP-1 (5′-CTG TTG TTG CTG TGG CTG ATA-3′ and 5′-CCG TCC ACA AGC AAT GAG T-3′), TIMP-2 (5′-GTA GTG ATC AGG GCC AAA G-3′ and 5′-TTC TCT GTG ACC CAG TCC AT-3′), iNOS (5′-ATG GCT TGC CCC TGG AAG TTT CTC-3′ and 5′-CCT CTG ATG GTG CCA TCG GGC ATC TG-3′), and COX-2 (5′-TTC ACC AGA CAG ATT GCT GGC-3′ and 5′-AGT CTG GAG TGG GAG GCA CTT G-3′). After amplification, the PCR reactants were electrophoresed in 1% agarose gels and visualized with ethidium bromide (EtBr, Sigma-Aldrich) staining. In a parallel experiment, glyceraldehyde-3-phosphate dehydrogenase (GAPDH, 5′-GAC CTG ACC TGC CGT CTA-3′ and 5′-AGG AGT GGG TGT CGC TGT-3′) was used as an internal control.

### Protein extraction, electrophoresis, and western blot analysis

Whole-cell protein extracts from HASMCs were prepared with cell lysis buffer (20 mM sucrose, 1 mM EDTA, 20 μM Tris–HCl, pH 7.2, 1 mM DTT, 10 mM KCl, 1.5 mM MgCl_2_, and 5 μg/ml aprotinin) for 30 min. The protein extracts were quantified using the Bio-Rad kit (Pierce Biotechnology, Rockford, IL, USA). For Western blot analysis, lysate proteins were resolved on sodium dodecyl sulfate (SDS)-polyacrylamide gel electrophoresis and transferred onto nitrocellulose transfer membranes (Schleicher & Schuell, Keene, NH, USA). Specific proteins were detected with an enhanced chemiluminescence (ECL) kit (Amersham Co., Arlington Heights, IL, USA) according to the recommended procedure. In a parallel experiment, cells were washed with ice-cold phosphate-buffered saline (PBS) and collected. Then cytoplasmic and nuclear proteins were prepared using NE-PER Nuclear and Cytoplasmic Extraction Reagents (Pierce Biotechnology). Antibodies against MMP-2, MMP-9, TIMP-1, TIMP-2, iNOS, COX-2, NF-κB p65, IκBα, nucleolin, and actin were purchased from Santa Cruz Biotechnology (Santa Cruz, CA, USA). The peroxidase-labeled donkey anti-rabbit immunoglobulin and peroxidase-labeled sheep anti-mouse immunoglobulin were purchased from Amersham Co.

### Immunofluorescence staining

HASMCs were cultured directly on glass coverslips in 6-well plates for 24 h to detect NF-κB p65 localization by immunofluorescence assay using a fluorescence microscope. After stimulation with TNF-α in the presence or absence of SSeo, the cells were fixed with 4% paraformaldehyde in PBS for 10 min at room temperature and permeabilized with 100% methanol for 10 min at 20°C. Polyclonal antibody against anti-NF-κB p65 was applied for 1 h followed by a 1 h incubation with fluorescein isothiocyanate (FITC)-conjugated donkey anti-rabbit IgG (Santa Cruz Biotechnology). After washing with PBS, nuclei were stained with 4,6-diamidino-2-phenyllindile (DAPI, Sigma-Aldrich) and fluorescence was visualized using a fluorescence microscope (Carl Zeiss, Oberkochen, Germany).

### Measurement of ROS generation

Intracellular accumulation of ROS was determined using the fluorescent probes 2′,7′-dichlorodihydrofluorescein diacetate (H2DCFDA, Sigma-Aldrich). Briefly, HASMCs were pretreated with 10 mM nacetylcysteine (NAC), ROS scavenger, or SSeo for 30 min before treatment with TNF-α (100 ng/ml) for 30 min. To measure intracellular ROS, the cells were incubated for 4 h at 37°C in PBS containing 20 mM H2DCFDA to label intracellular ROS. ROS production in the cells was monitored with a flow cytometer (FACS Calibur; Becton Dickinson, San Jose, CA, USA) using the Cell-Quest pro software [42].

### Nitrite measurement

Concentrations of nitric oxide (NO) in the culture supernatants were determined by measuring nitrite, a stable oxidation product of NO, using Griess reagent (Sigma-Aldrich). Briefly, the supernatant from cell cultures was collected, mixed with an equal volume of Griess reagent, and incubated at room temperature for 10 min. NaNO_2_ was used to generate a standard curve, and nitrite production was determined by measuring optical density at 550 nm [43].

### Determination of prostaglandin E_2_ (PGE_2_) production

To determine the levels of PGE_2_, an aliquot of culture medium supernatant was collected and the concentration (pg/ml) of PGE_2_ in the cell culture medium was calculated by based on the concentrations of the standard solution using a PGE_2_ enzyme-linked immunosorbent assay (ELISA) kit following the manufacturer’s instructions (Cayman Chemical Co., Ann Arbor, MI, USA).

### Cell invasion assay

The cell migration assay was performed using the Transwell system (Corning Costar, Cambridge, MA, USA). Briefly, HASMCs were resuspended in 100 μL of medium and placed in the upper part of the Transwell plate. The cells were incubated for 8 h, fixed with methanol, and then stained with haematoxylin for 10 min followed by eosin Y (Sigma-Aldrich). HASMCs on the upper surface of the filter were mechanically removed by wiping with a cotton swab, and the migrated cells were determined by counting the cells (three fields of each triplicate filter) that migrated to the lower side of the filter using an inverted microscope.

### Statistical analysis

Data are expressed as the mean ± standard deviation (SD) values. One-way analysis of variance (ANOVA) was used for comparisons in the experiments with multiple time points and concentrations. When ANOVA indicated statistical significance, Duncan’s multiple range test was used to determine which means were significantly different. A probability value of p < 0.05 was used as the criterion for statistical significance.

## Results

### SSeo inhibits TNF-α-induced MMP-9 activation in HASMCs

**Figure 1:**
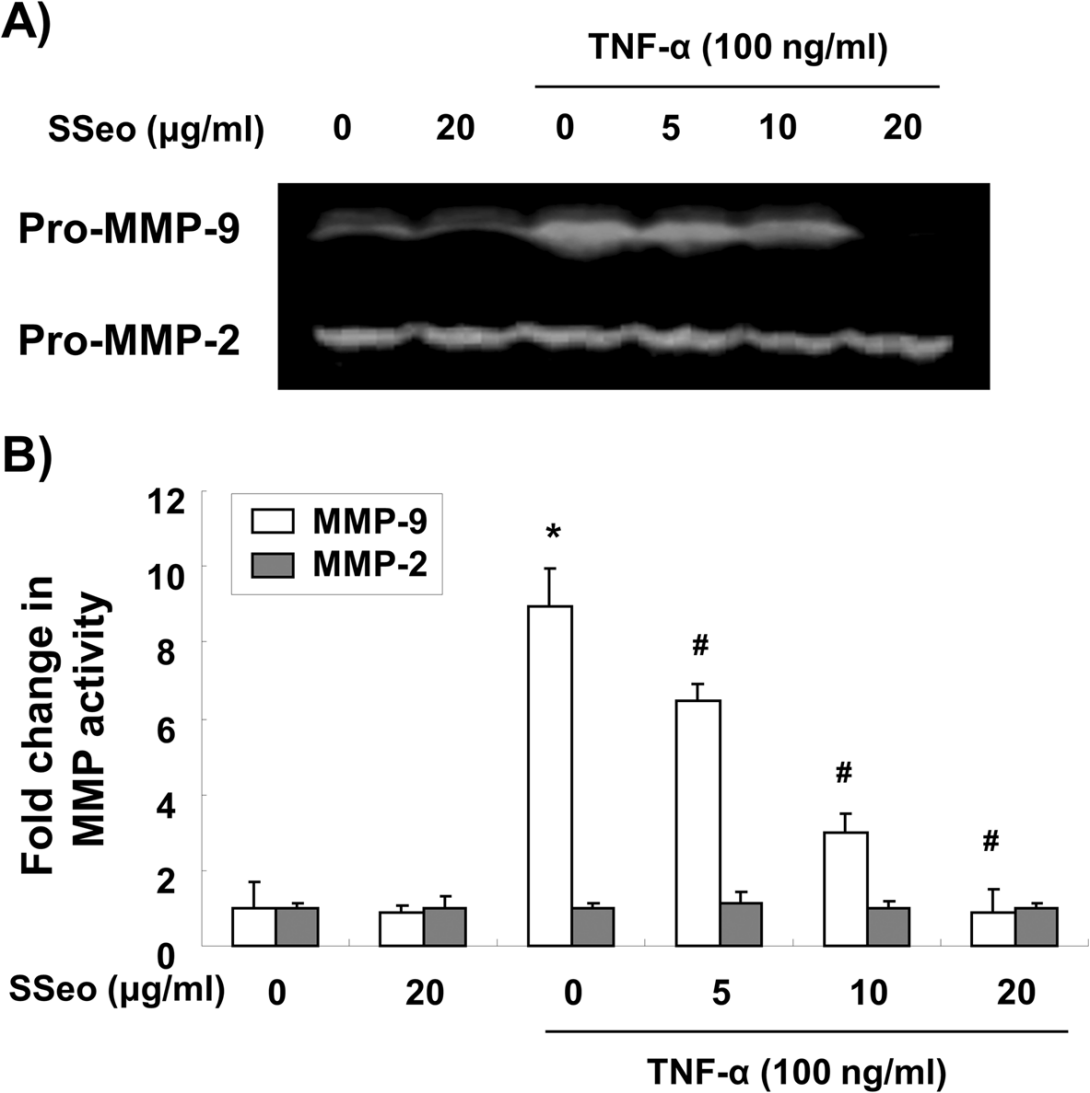
**Inhibition of TNF-α-induced MMP-9 activation by SSeo in HASMCs. (A)** HASMCs were pretreated for 1 h with different concentrations of SSeo, followed by incubation with TNF-α for 24 h. The culture medium was collected and analyzed for gelatinolytic activity by zymography. **(B)** *In vitro* activity of MMP-2 and −9 in cell culture supernatant was measured using a MMP gelatinase activity assay kit. The biotinylated gelatinase substrates were cleaved by active MMPs in the samples, and the fragments were added to a biotin-binding plate. The digested but unbound fragments were removed by washing. Data are mean ± SD from three independent experiments and are presented as fold change compared with untreated control cells (*, *p* < 0.05 *vs.* untreated control; ^#^, *p* < 0.05 *vs.* TNF-α-treated HASMCs).

HASMC migration is one of the most important characteristics in atherosclerotic diseases, and the molecular mechanisms have been extensively studied. Many studies indicate that MMPs may participate in the development of atherosclerosis. Among them, an increase in MMP-9 production could contribute to an invasive HASMC phenotype [6-8]; thus, we investigated the effect of SSeo on TNF-α-induced MMP-9 activation. HASMCs were treated with TNF-α (100 ng/ml) in the presence or absence of various concentrations of SSeo for 24 h. At the end of the incubation, media were collected and assayed for MMP activity using gelatin zymography. As shown in Figure 1A, although, MMP-9 had very weak activity, and MMP-2 had a high secretion level in the control condition media, treatment with TNF-α increased the level of MMP-9 secretion, but had no effect on the level of MMP-2 secretion. However, SSeo significantly diminished TNF-α-induced MMP-9 secretion in a concentration-dependent manner. Additionally, s similar result was observed in the MMP-9 matrix degradation activity assay but not MMP-2 using the MMP gelatinase activity assay kit (Figure 1B).

### Effect of SSeo on HASMCs viability

**Figure 2:**
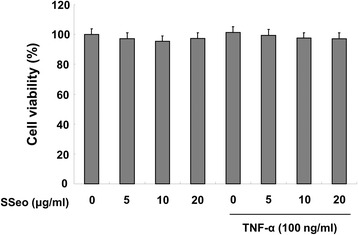
**Effects of SSeo on cell viability of HASMCs.** The cells were pretreated for 1 h with the indicated concentrations of SSeo, followed by incubation with TNF-α for 24 h. Cell viability was measured using the MTT assay. Each point represents the mean ± SD of three independent experiments.

HASMCs were exposed to various concentrations of SSeo for 24 h with or without TNF-α, and cellular toxicity was analyzed using the MTT assay. Treatment of HASMCs with the indicated concentrations of SSeo used to inhibit MMP-9 activation did not cause any significant change in cell viability (Figure 2). These results clearly indicate that inhibiting MMP-9 activation in TNF-α-stimulated HASMCs was not due to a cytotoxic action of SSeo.

### SSeo reduces TNF-α-induced MMP-9 expression in HASMCs

**Figure 3:**
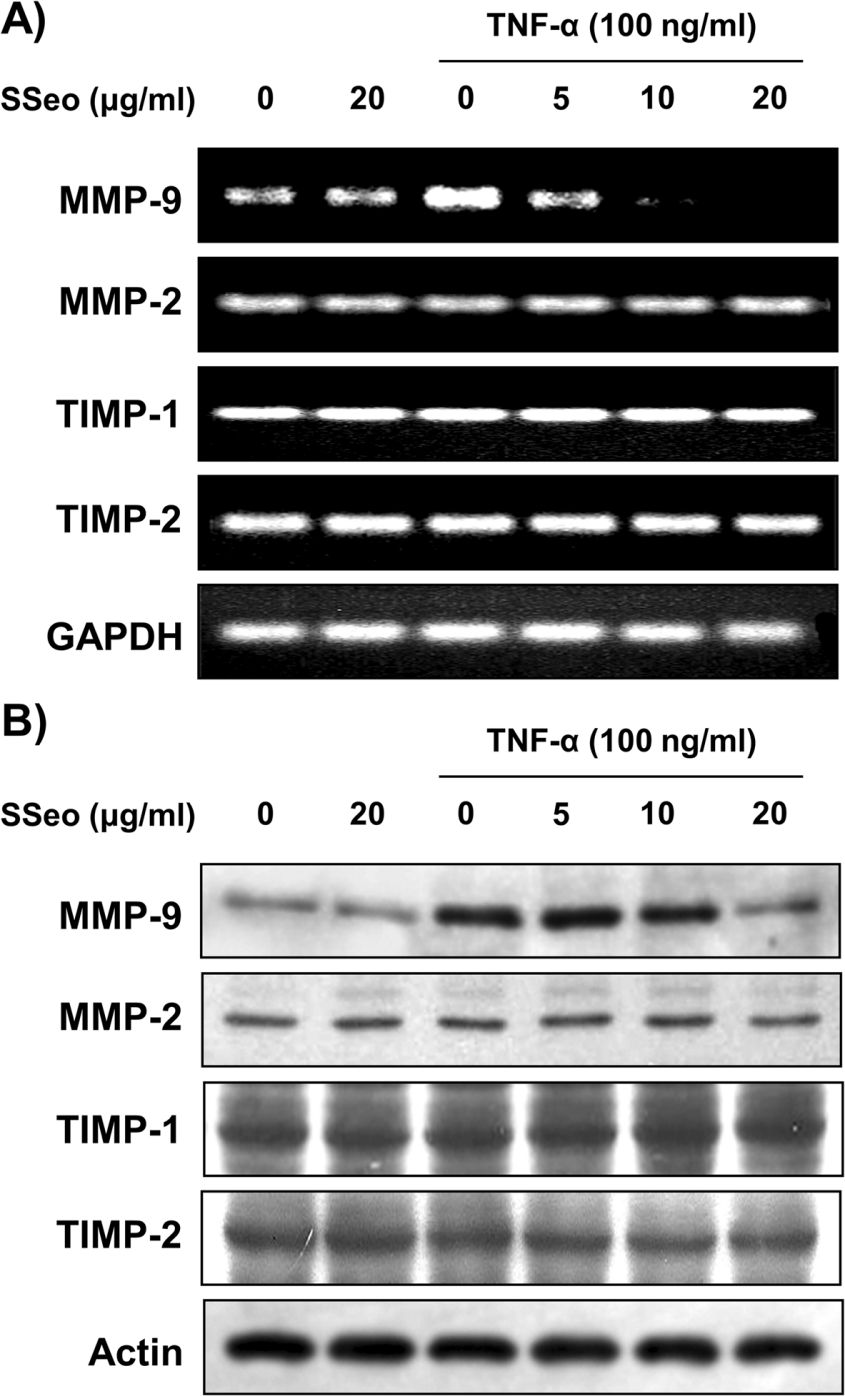
**Inhibition of TNF-α-induced MMP-9 mRNA and protein expression by SSeo in HASMCs. (A)** Total RNA was isolated from cells grown under the same conditions as Figure 1 and reverse-transcribed. Resulting cDNAs were then subjected to PCR. The reaction products were run on 1% agarose gel electrophoresis and visualized by EtBr staining. **(B)** The cells were sampled, lysed, and 30–50 μg of protein was separated by SDS-polyacrylamide gel electrophoresis. Western blotting was then performed using the indicated antibodies and an ECL detection system. GAPDH and actin were used as the internal controls for the RT-PCR and Western blot analyses, respectively.

After the inhibition activity of SSeo on MMP-9 was confirmed, RT-PCR and Western blot analyses were performed to determine the effects of SSeo on the levels of MMP-9 in TNF-α-treated HASMCs. Figure 3 illustrates that SSeo concentration-dependently reduced TNF-α-induced MMP-9 expression at both the transcriptional and translational levels, but did not affect MMP-2 levels. Next, to determine the effects of SSeo on MMPs-related endogenous inhibitors, the levels of TIMP-1 and −2 were examined. As shown in Figure 3, the levels of TIMP-1 and −2 mRNA and protein showed no significant changes in HASMCs treated with or without TNF-α and SSeo. These results suggest that SSeo suppresses TNF-α-induced MMP-9 activity by inhibiting MMP-9 transcription level in HASMCs, which was not associated with TIMPs expression.

### SSeo suppresses TNF-α-induced HASMCs invasion

**Figure 4:**
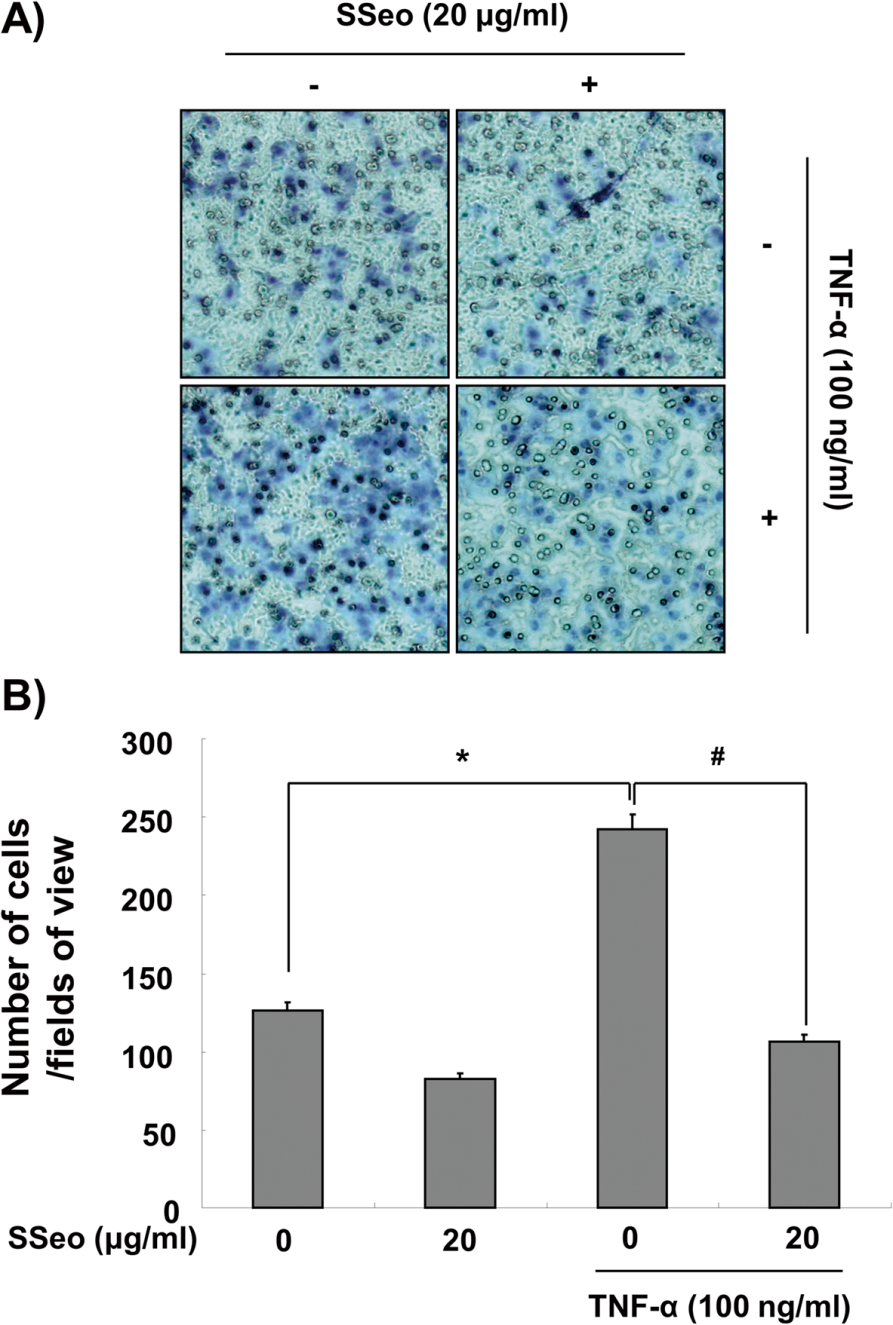
**Effects of SSeo on TNF-α-induced HASMC migration. (A)** Invasiveness of the cells was determined by measuring their ability to pass through a layer of a Matrigel-coated filter. Following treatment with TNF-α (100 ng/ml) in the presence or absence of SSeo (20 μg/ml) for 8 h, cells on the bottom side of the filter were fixed, stained and counted. **(B)** Data from three independent experiments are expressed as overall mean ± SD. Significance was determined using Student’s t-test (*p < 0.05 vs. untreated control; ^#^ *p* < 0.05 vs. TNF-α-treated HASMCs).

As up-regulation of MMP-9 expression by TNF-α contributes to invasion of HASMCs [7,8] and SSeo decreased MMP-9 activity in TNF-α treated HASMCs (Figure 1), an *in vitro* invasion assay was used to investigate the inhibitory effects of SSeo on the invasive potency of HASMCs by a Matrigel invasion assay. To measure the invasion rate, we counted migrated HASMCs that penetrated the Matrigel and moved to the backside of the Transwell membrane. As shown in Figure 4, treatment with TNF-α significantly increased HASMC invasion, and treatment with SSeo alone partially decreased HASMC invasion compared to that of the control. However, pretreatment with SSeo significantly diminished the TNF-α-induced cell invasion to lower levels than those observed in the control, indicating that MMP-9 suppression may play a central role in the inhibitory effect of SSeo on TNF-α-induced HASMC migration.

### SSeo attenuates the TNF-α-induced inflammatory response in HASMCs

**Figure 5:**
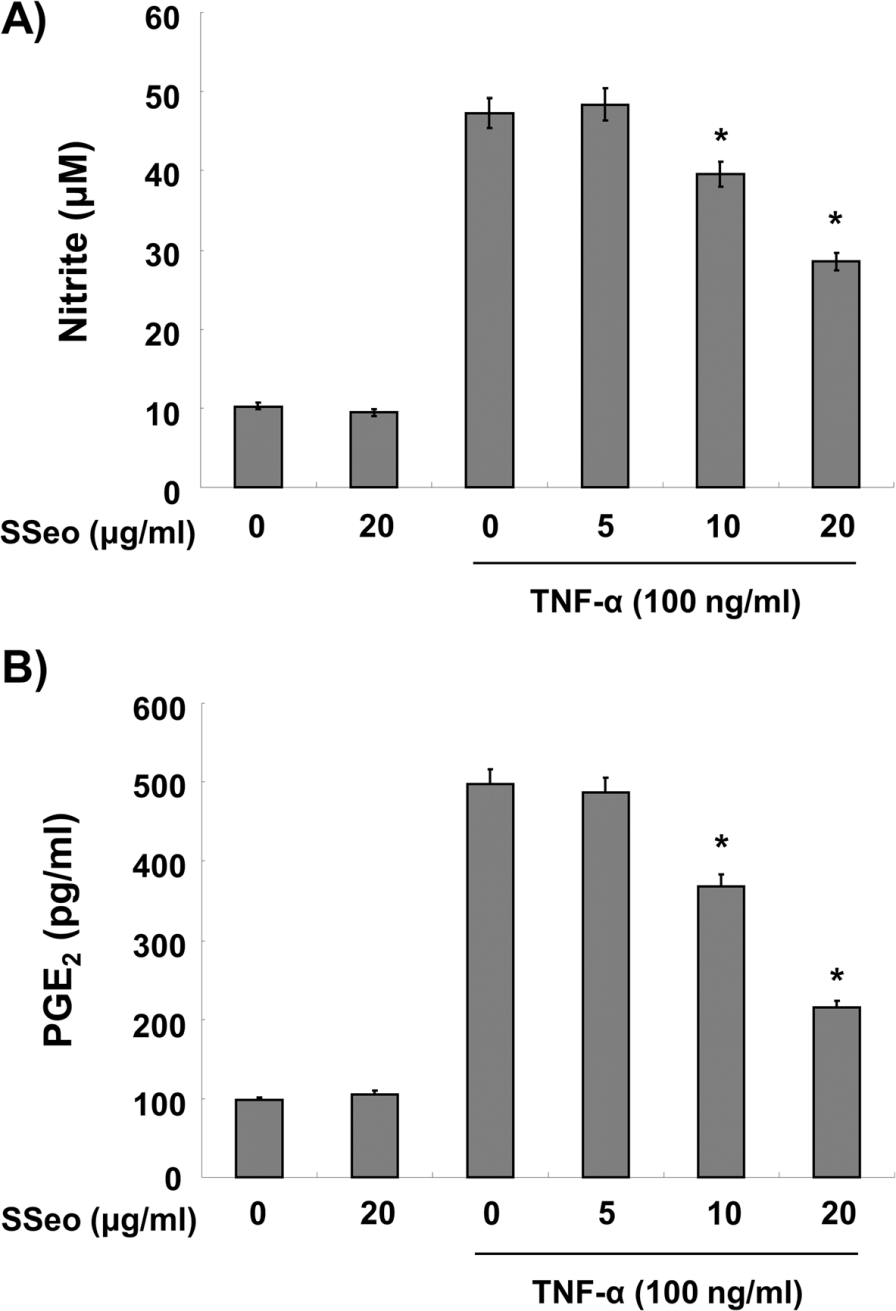
**Inhibition of NO and PGE**_**2**_ **production by SSeo in TNF-α-treated HASMCs.** HASMCs were pretreated with various concentrations of SSeo for 1 h before incubation with TNF-α (100 ng/ml) for 24 h. **(A)** Nitrite content was measured using the Griess reagent and **(B)** PGE_2_ concentration was measured in culture media using a commercial ELISA kit. Values represent the mean ± SD of three independent experiments. We assessed differences between mean values by the Student’s *t*-test. *p < 0.05 indicates significant differences from the TNF-α-treated group.

**Figure 6:**
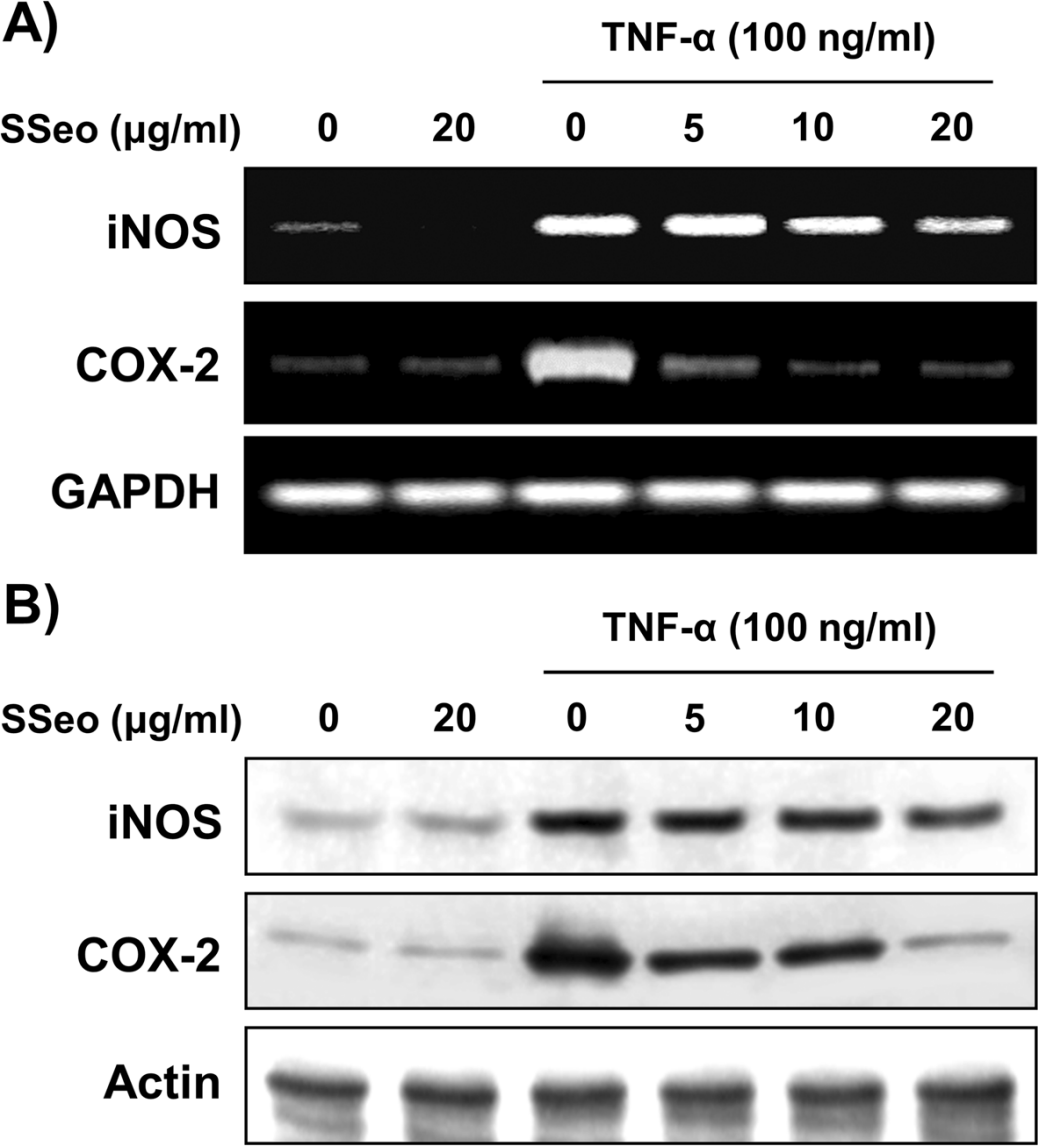
**Inhibition of iNOS and COX-2 expression by SSeo in TNF-α-treated HASMCs. (A)** HASMCs were pretreated with the indicated concentrations of SSeo 1 h prior to incubation with TNF-α (100 ng/ml) for 6 h. Total RNA was prepared for RT-PCR analysis of iNOS and COX-2 gene expression. The reaction products were run on 1% agarose gel electrophoresis and visualized by EtBr staining. **(B)** After TNF-α treatment for 24 h, cell lysates were prepared and Western blotting was performed using anti-iNOS and anti-COX-2 antibodies, and an ECL detection system. GAPDH and actin were used as the internal controls of RT-PCR and Western blot analysis, respectively.

Because it is well known that activation of NF-κB induces the expression of pro-inflammatory mediators [14,15], HASMCs were stimulated with TNF-α for 24 h in the presence and absence of SSeo to examine the inhibitory effect of SSeo on the TNF-α-induced inflammatory response. Treatment of HASMCs with TNF-α alone elevated the levels of pro-inflammatory mediators such as NO and PGE_2_ compared to those in the control (Figure 5). However, treating the cells with TNF-α in the presence of SSeo abrogated the ability of TNF-α to induce pro-inflammatory mediator release. As expected, SSeo also attenuated TNF-α-induced iNOS and COX-2 mRNA and protein expressions, to levels comparable to those of the control (Figure 6). These results indicate that the reduced expression of pro-inflammatory enzymes at the transcriptional level contributed to the inhibitory effect of SSeo on TNF-α-induced NO and PGE_2_ production.

### SSeo blocks TNF-α-induced ROS formation in HASMCs

**Figure 7:**
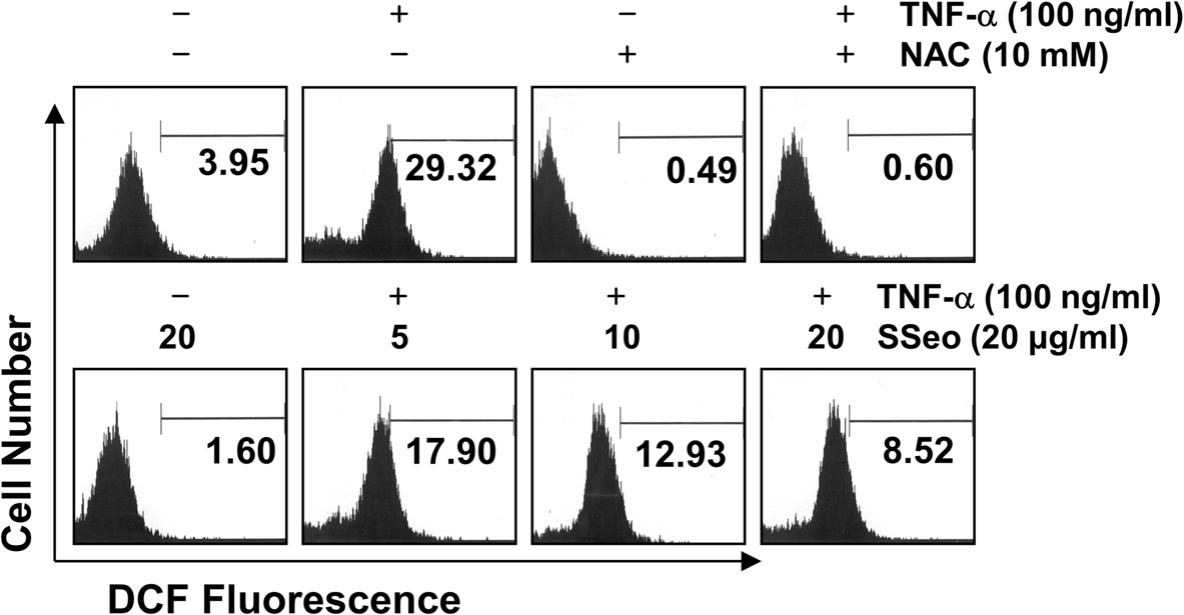
**Effects of SSeo on TNF-α-induced intracellular ROS generation in HASMCs.** HASMCs were treated with NAC (10 mM) or the indicated concentrations of SSeo for 30 min before treatment with TNF-α (100 ng/ml) for 30 min. The cells were incubated with 20 mM H2DCFDA at 37°C for 30 min, and ROS generation was measured using a flow cytometer. Each point represents the mean of two independent experiments.

Several studies have reported that TNF-α-mediated activation of NF-κB leads to enhanced ROS production, as a common second messenger, and NF-kB activation, thereby contributing to sustained oxidant production during chronic inflammation [17,20]. Therefore, the level of intracellular ROS generation was assessed to determine whether SSeo can reduce the level of TNF-α-induced oxidative stress in HASMCs using flow assisted cytometry analysis. As shown in Figure 7, TNF-α significantly enhanced ROS production, while pretreatment with SSeo considerably reversed TNF-α-induced cellular ROS production in a concentration-dependent manner, indicating that SSeo is capable of abrogating the increased ROS levels observed in TNF-α-treated HASMCs. Treatment with SSeo alone also decreased ROS levels when compared with untreated control cells.

### SSeo inhibits TNF-α-induced nuclear translocation of NF-κB in HASMCs

**Figure 8:**
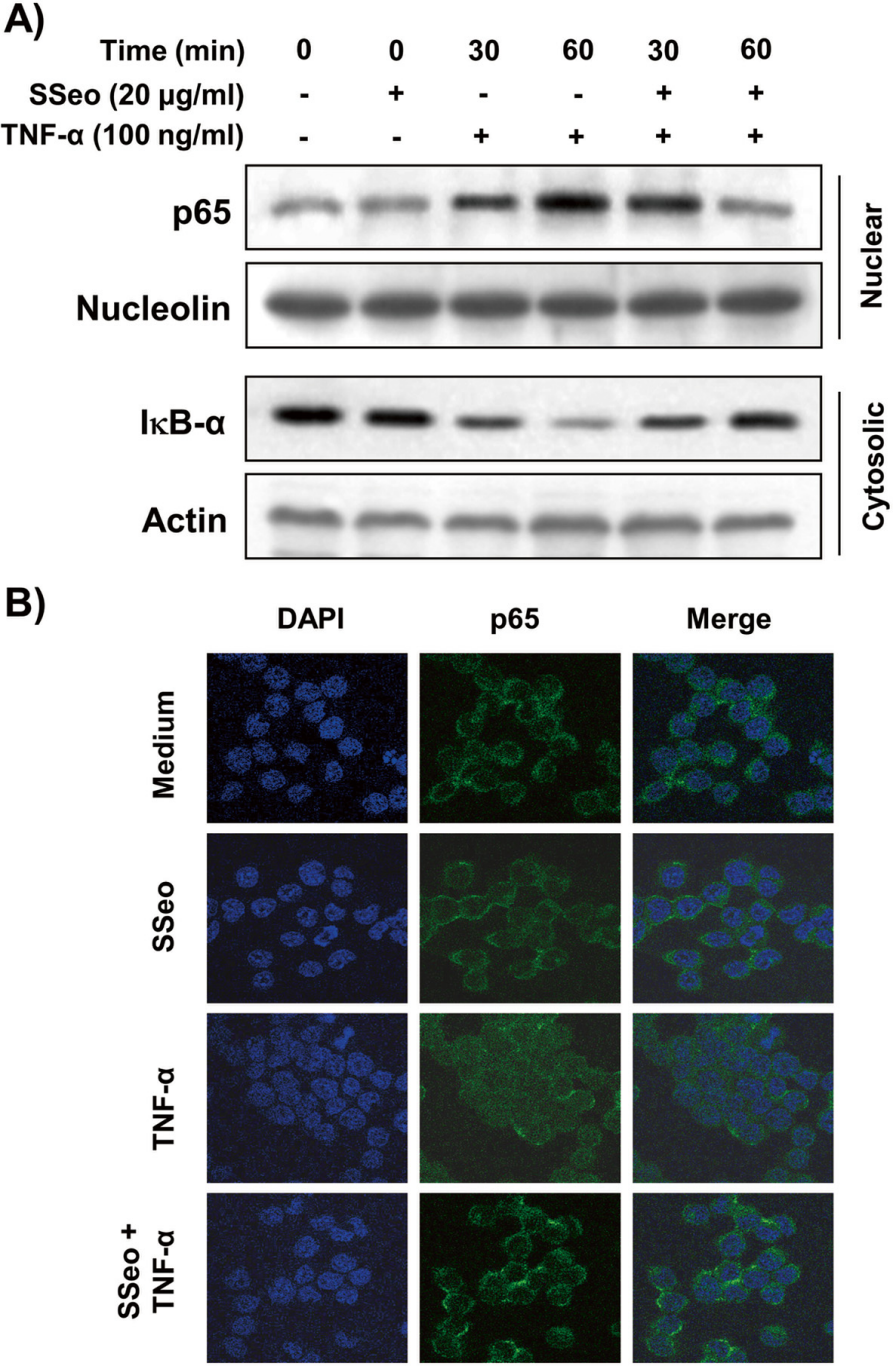
**Inhibition of NF-κB nuclear translocation by SSeo in TNF-α-stimulated HASMCs. (A)** Cells were pretreated with SSeo (20 μg/ml) for 1 h before TNF-α treatment (100 ng/ml) for the indicated times. **(A)** Cytosolic and nuclear proteins were run on 10% SDS-polyacrylamide gels followed by Western blotting using anti-NF-κB p65 and anti-IκB-α antibodies, and an ECL detection system. Nucleolin and actin were used as internal controls for the nuclear and cytosolic fractions, respectively. **(B)** The cells were pretreated with SSeo (20 μg/ml) for 1 h before TNF-α treatment (100 ng/ml). After a 1 h incubation, localization of NF-κB p65 was visualized with fluorescence microscopy after immunofluorescence staining with anti-NF-κB p65 antibody (green). Cells were also stained with DAPI to visualize nuclei (blue). Results are representative of those obtained from three independent experiments.

Many studies have reported that TNF-α-induced NF-κB activation is involved in upregulating MMP-9 transcriptional activity; thus, we determined whether the inhibitory effect of SSeo on TNF-α-induced activation of MMP-9 is mediated through suppression of NF-κB signaling by measuring the nuclear translocation of NF-κB. Western blot analyses using cytosolic and nuclear fractions showed that treatment of TNF-α enhanced nuclear accumulation of NF-κB proteins, concomitantly with degradation of IκB-α in cytosol. However, pretreatment of HASMCs with SSeo prior to TNF-α stimulation significantly prevented nuclear accumulation of NF-κB, and TNF-α-induced IκB-α degradation was obviously blocked by pretreatment with SSeo (Figure 8A). The immunofluorescence images also revealed that nuclear accumulation of NF-κB p65 was not induced in cells after treatment with SSeo alone in the absence of TNF-α stimulation; however, that was strongly induced after stimulation of HASMCs with TNF-α, and the shift in NF-κB p65 to the nucleus was completely abolished after pretreating the cells with SSeo (Figure 8B). These results suggest that the inhibitory effect of SSeo on TNF-α-induced MMP-9 expression is related to inactivation of NF-κB by preventing IκB-α degradation.

## Discussion

In the present study, we investigated the effects of SSeo on TNF-α-induced MMP-9 activation and cell invasion in HASMCs. Our data demonstrate that SSeo effectively inhibited the increased levels of secretion and expression of MMP-9, and the nuclear translocation of the NF-κB in TNF-α-stimulated HASMCs. We also found that SSeo has the ability to suppress the TNF-α-induced release of intracellular ROS and inflammatory mediators such as NO and PGE_2_.

A number of studies have demonstrated that the migration of VSMCs from the tunica media to the subendothelial region is a key event in the development and progression of atherosclerosis after vascular injury [1,2]. Recent studies have identified enhanced expression of MMPs in atherosclerotic lesions and their contribution to weakening of the vascular wall by degrading all kinds of ECM proteins. Among MMPs, MMP-9, which is induced in response to many stimulants, may specifically contribute to the pathogenesis of atherosclerosis by facilitating migration of VSMCs [3,4]. These processes are also promoted by inflammatory mediators and cytokines, as well as cell-cell contact signaling. These observations indicate that the development of therapeutic drugs specifically targeting MMP-9 and inhibition of VSMC migration may be useful for preventing atherosclerotic lesion progression. Our results demonstrated that pretreatment with SSeo significantly inhibited TNF-α-induced MMP-9 secretion by suppressing transcriptional activity of the MMP-9 gene in HASMCs (Figures 1 and 3). In general, the activity of MMPs is tightly controlled by transcriptional activation, by a complex proteolytic activation cascade, and by an endogenous system of TIMPs. TIMPs inhibit MMPs by forming stoichiometric complexes to regulate matrix turnover [44]. However, the transcriptional and translational levels of TIMP-1 and −2 remained unchanged in HASMCs treated with TNF-α alone or in a combined treatment with SSeo at the concentrations tested (Figure 3), suggesting that SSeo suppressed MMP-9 expression by diminishing its gene transcription without abolishing TIMP-1 and −2 expression.

Many studies have identified the signaling mechanisms underlying the regulation of transcription factors that are involved in regulating MMP-9 expression. Most of all, a functional NF-κB site occurs in the proximal stimulatory region of the MMP-9 promoter [11,12,15], and a previous study demonstrated that transient overexpression of IκBα in VSMCs only partially impairs upregulation of MMP-9, suggesting that NF-κB might play a simple permissive role [45]. Thus, we focused here on defining the role played by the NF-κB transcription factor in the downregulation of MMP-9 activity by SSeo in TNF-α-stimulated HASMCs. In general agreement with previous reports [10-12,14,15], the majority of intracellular NF-κB p65 translocated from the cytosol to the nucleus following treatment with TNF-α (Figure 8). However, the levels of NF-κB p65 in the nucleus decreased significantly following pretreatment with SSeo, and TNF-α-induced IκB-α degradation was also significantly reversed by SSeo. Furthermore, data obtained from the Matrigel migration assay indicated that SSeo significantly inhibited TNF-α-induced migration potential of HASMCs (Figure 4). These results led us to conclude that SSeo inhibits TNF-α-induced nuclear translocation of NF-κB, thereby suppressing activation and protein expression of MMP-9, resulting in decreased HASMC migration.

In contrast, oxidative stress is a state in which excess ROS overwhelms endogenous antioxidant systems. Several studies have indicated that ROS are implicated in the activation of NF-κB, and inflammatory mediators are also implicated in the production of ROS. Moreover, previous results indicate that inflammatory mediators, which strongly influence the production of atherosclerotic plaque, stimulate VSMC migration from the intima to the media [16,17]. In addition, oxidative stress affects injured vessels, which develops into inflammation. In contrast, inflammation can also increase ROS on atherosclerotic lesions to regulate cellular reaction such as VSMC proliferation and migration [16,46], suggesting that atherosclerosis is a chronic inflammatory disease associated with increased oxidative stress in the VSMCs. This vicious cycle leads not only to cardiovascular disease but also myocardial infarction, stroke, and heart failure [47]. Thus, reducing oxidative stress and production of inflammatory mediators is important to control atherosclerosis. In our experiments, SSeo pretreatment blocked TNF-α-stimulated production of ROS, indicating that SSeo could scavenge radicals (Figure 7). Moreover, SSeo effectively inhibited TNF-α-induced NO and PGE_2_ synthesis (Figure 5) and that this suppression was consistently correlated with downregulation of iNOS and COX-2 expression (Figure 6). Therefore, we propose that the inhibitory effect of SSeo on MMP-9 expression and NF-κB activation may be due to its antioxidant and anti-inflammatory properties.

## Conclusions

Collectively, our data reveal for the first time that SSeo, an essential oil purified from Schisandrae semen, strongly suppressed TNF-α-induced MMP-9 expression and migration of HASMCs by inhibiting activation of the NF-κB signaling pathway. SSeo also effectively downregulated TNF-α-induced production of ROS and inflammatory mediators in HASMCs. Although future studies on its regulation of VSMC proliferation and migration *in vivo*, as well as the detailed mechanisms are needed, these observations indicate that SSeo might be useful as a therapeutic agent for preventing and/or treating vascular disorders related to VSMC migration.
